# Heterogeneous Expression of *Arabidopsis* Subclass II of SNF1-Related Kinase 2 Improves Drought Tolerance via Stomatal Regulation in Poplar

**DOI:** 10.3390/life14010161

**Published:** 2024-01-22

**Authors:** Borislav Horvat, Yuhei Shikakura, Misato Ohtani, Taku Demura, Akira Kikuchi, Kazuo N. Watanabe, Taichi Oguchi

**Affiliations:** 1 Degree Program in Life and Earth Science, Graduate School of Science and Technology, University of Tsukuba, Tsukuba 305-8572, Ibaraki, Japan; 2 Department of Integrated Biosciences, Graduate School of Frontier Sciences, The University of Tokyo, 5-1-5 Kashiwanoha, Kashiwa 277-8562, Chiba, Japan; 3 RIKEN Center for Sustainable Resource Science, 1-7-22 Suehiro-cho, Tsurumi-ku, Yokohama 230-0045, Kanagawa, Japan; 4 Center for Digital Green-Innovation, Nara Institute of Science and Technology, Ikoma 630-0192, Nara, Japan; 5 Institute of Life and Environmental Sciences, University of Tsukuba, Tsukuba 305-8572, Ibaraki, Japan; 6 Tsukuba Plant Innovation Research Center, University of Tsukuba, Tsukuba 305-8572, Ibaraki, Japan

**Keywords:** poplar, SnRK2, stomatal regulation, osmotic stress, abiotic stress, ABA signal transduction

## Abstract

Abscisic acid (ABA) is the most important phytohormone involved in the response to drought stress. Subclass II of SNF1-related kinase 2 (SnRK2) is an important signaling kinase related to ABA signal transduction. It regulates the phosphorylation of the target transcription factors controlling the transcription of a wide range of ABA-responsive genes in *Arabidopsis thaliana*. The transgenic poplars (*Populus tremula* × *P. tremuloides*, clone T89) ectopically overexpressing *AtSnRK2.8*, encoding a subclass II SnRK2 kinase of *A. thaliana*, have been engineered but almost no change in its transcriptome was observed. In this study, we evaluated osmotic stress tolerance and stomatal behavior of the transgenic poplars maintained in the netted greenhouse. The transgenic poplars, line S22, showed a significantly higher tolerance to 20% PEG treatment than non-transgenic controls. The stomatal conductance of the transgenic poplars tended to be lower than the non-transgenic control. Microscopic observations of leaf imprints revealed that the transgenic poplars had significantly higher stomatal closures under the stress treatment than the non-transgenic control. In addition, the stomatal index was lower in the transgenic poplars than in the non-transgenic controls regardless of the stress treatment. These results suggested that *AtSnRK2.8* is involved in the regulation of stomatal behavior. Furthermore, the transgenic poplars overexpressing *AtSnRK2.8* might have improved abiotic stress tolerance through this stomatal regulation.

## 1. Introduction

Plants, being inherently sessile organisms, are confronted with the inescapable reality of stress, as their fixed position renders them unable to evade or seek refuge from adverse environmental conditions. To survive challenging environmental cues, plants have intricately evolved a repertoire of mechanisms aimed at both tolerating and resolutely resisting unfavorable conditions. Furthermore, in order to develop climate-resilient crops and forestry trees adaptable to environmental stresses as a countermeasure against the worsening climate change, it is important to accumulate plant molecular and physiological knowledge of plant environmental responses [1]. Abscisic acid (ABA) is a phytohormone involved in various physiological processes in plants, regulating plant growth, development, and responses to stresses [2]. ABA is arguably the most important phytohormone involved in plant response to drought stress [3,4]. The ABA-dependent response to drought stress is well-researched, and in *A. thaliana*, it involves a couple of key players.

SNF1-related protein kinase (SnRK) constitutes a family of serine/threonine protein kinases in plants [5]. The members of the SnRK family are divided into SnRK1, SnRK2, and SnRK3 subfamilies by differences found in the C-terminal domains [6]. The SnRK1 subfamily is the plant orthologue of yeast SNF1 (sucrose non-fermenting 1) and mammalian AMP-activated protein kinase (AMPK), which acts as a sensor molecule for carbohydrate metabolism [5]. Plant SnRK1s have functions similar to yeast SNF1 and mammalian AMPK (Figure S1) [5]. On the other hand, the SnRK2 and SnRK3 subfamilies are unique in plants (Figure S1) [7]. Both SnRK2 and SnRK3 have been shown to play a role in abiotic stress response [8]. SnRK3s interact with calcineurin B-like calcium sensor proteins (CBLs) via NAF domain, one of the functional conserved domains, and regulate plant response to abiotic stress in a Ca^2+^ signaling dependent manner [9]. SnRK2s are categorized into three subclasses (Figures S1 and S2) [8]. They all have functions in abiotic stress response, but differ in their dependence on ABA (Figure S1) [8]. In other words, subclass II and subclass III SnRK2 are involved in regulating stress responses in an ABA-dependent manner, while subclass I SnRK2 is involved in regulating stress responses in an ABA-independent manner (Figure S1) [8]. ABA binds to specific receptor proteins known as PYR (PYRABACTIN RESISTANCE)/PYL (PYR-like)/RCAR (REGULATORY COMPONENT OF ABA RECEPTOR) (Figure S3) [10,11]. ABA-bound PYR/PYL/RCAR interacts with PP2C (clade A protein phosphatase type 2C), such as ABI1 and ABI2, which binds SnRK2 kinases and inhibits their activity (Figure S3) [10,11]. This interaction facilitates the activation of SnRK2 kinases by the autophosphorylation or activation by other protein kinases, such as Raf-like kinases [12,13]. Through the phosphorylation of downstream proteins, activated subclass II and III SnRK2s mainly modulate the transcription of stress responsive genes and proteins related to stomatal closure, respectively (Figure S3) [8,14].

Based on the whole genome sequence information, there are 10 SnRK2 kinases in *A. thaliana* subclass I (SnRK2.1, 2.4, 2.5, 2.9, and 2.10), subclass II (SnRK2.7, and 2.8), and subclass III (SnRK2.2, 2.3, and 2.6) [6,15]. Subclasses II and III are known to have functions in ABA signal transduction [15]. In particular, an *Arabidopsis* subclass II SnRK2, *AtSnRK2.8* (also known as AtSRK2C; TAIR: AT1G78290) regulates the transcription of various stress-responsive genes, and its homogeneous overexpression in the plant exhibits extremely strong tolerance to environmental stresses [16]. In contrast to *A. thaliana*, there are few reports on abiotic stress response pathways in woody plants. *Populus* (poplars) are important trees for both afforestation and industrial plantation because of their rapid growth and biomass production abilities in mid-latitude regions [17]. In addition, *Populus* is known as the model organism for research in woody plants [18,19,20]. With the recent developments in whole genome analysis technology, whole genomes of several *Populus* species have been reported. Twelve SnRK2 kinases were identified in *P. trichocarpa*: subclass I (SnRK2.1, 2.2, 2.3, and 2.4), subclass II (SnRK2.5, 2.6, 2.7, 2.8 and 2.9) and subclass III (SnRK2.10, 2.11 and 2.12) (Figure S2) [21]. Transgenic *Arabidopsis* ectopically overexpressing subclass II SnRK2 genes (*PtriSnRK2.5*, *PtriSnRK2.7*) derived from *P. trichocarpa* were reported to have a slightly improved stress tolerance, but no significant changes in the expression of stress-responsive genes was observed [22]. Similarly, a slight improvement in stress tolerance has been reported in transgenic poplar (*P. tremula* × *P. tremuloides*, clone T89) ectopically overexpressing *AtSnRK2.8* but no changes in the expression of stress-responsive genes were observed [23,24]. These reports suggested that although the sequence information of subclass II SnRK2 kinases were conserved, their functions in *Arabidopsis* and poplar may vary. In other words, it can be considered that poplar subclass II SnRK2 kinases might have different regulatory outputs beyond transcriptional regulation.

In this study, we considered stomatal regulations as an alternative criterion in evaluating the SnRK2 kinases in poplar. Subclass III SnRK2 kinases have been shown to function in the regulation of stomatal closure through direct phosphorylation of Slow anion channel 1 (SLAC1), an ion channel protein on the stomata membrane in *Arabidopsis* [25]. In addition, subclass III SnRK2 kinases have been linked with the regulation of stomatal differentiation via the phosphorylated activation of the basic helix-loop-helix (bHLH) transcription factor SPEECHLESS (SPCH) [26]. Therefore, we conducted quantitative monitoring of injury and leaf conductance in response to mild osmotic stress in young transgenic poplar trees. Furthermore, we evaluated stomatal opening and the number of stomata post-osmotic stress treatment, considering the novel abiotic stress response mechanism of subclass II SnRK2 kinases via stomatal regulation in poplar.

## 2. Materials and Methods

### 2.1. Plant Materials and Cultivation Conditions

**Figure 1:**
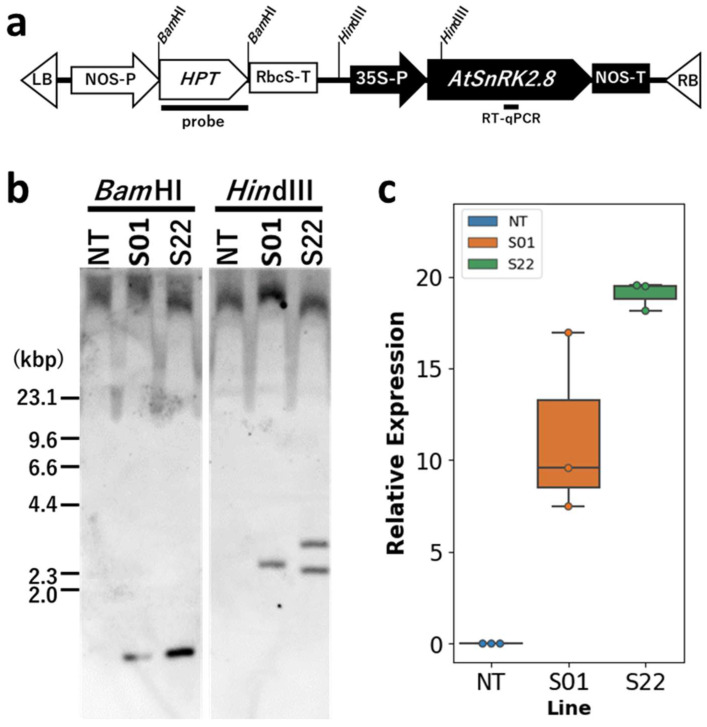
T-DNA introduced into transgenic lines and their genetical characteristics. (**a**) The scheme of T-DNA introduced into transgenic poplars. Recognition sites of restriction enzyme sites used for DNA gel blot analysis were shown at the top of the scheme. At the bottom of the scheme, the positions of the probes used for the DNA gel blot analysis and the targeted sequences for RT-qPCR analysis are shown. (**b**) DNA gel blot analysis of the genomic DNA of the transgenic poplars grown in vitro. The transgenic and non-transgenic poplar genomic DNAs were digested by *Bam*HI or *Hind*III and subjected to the hybridization with DIG-labeled HPT fragments. (**c**) Expression levels of the transgene in the transgenic poplars grown in vitro. The RT-qPCR analysis was performed for *AtSnRK2.8*. The relative expression levels of *AtSnRK2.8* were calculated by the *PtelF4a* expression. Error bars indicate standard errors (*n* = 3).

The genetically independent transgenic poplar lines S01 and S22 generated previously were used in this study [27]. The transgenic poplars were expressing a subclass II of the SnRK2 gene, *AtSnRK2.8,* derived from *A. thaliana* and *hygromycin phosphotransferase* gene (*HPT*) (Figure 1a). The hybrid aspen clone, line T89 (*P. tremula* × *P. tremuloides*), was used as non-transgenic control. A scheme outlining the poplar transformation method was shown in Supplementary Figure S4.

The transgenic and non-transgenic poplars were subcultured on a half-strength Murashige and Skoog (MS) medium, including vitamins (Duchega, Haarlem, The Netherlands) with Phytagel^®^ 2.7 g/L (Sigma Aldrich, St. Louis, MO, USA) under constant 25 °C, with light condition: 80 μmol/m^2^/s, 16/8 h light/dark photoperiod.

One-month-old in vitro plants were transferred to small square pyramidal pots (4.5 cm × 4.5 cm × 12.5 cm), filled with Genki-Kun 1-Gou (Zen-noh, Tokyo, Japan), and acclimatized to the cultivation room condition at 25 °C, 16 h light (60 µmol/m^2^/s by fluorescent tube (FL40SS, Panasonic, Osaka, Japan))/8 h dark. After about four-weeks of acclimatization in the cultivation room, the plants were then transferred to a netted greenhouse located in Tsukuba, in the Northern Kanto Plain, and at the center of the main island of Japan (36°07′ N, 140°06′ E) on 30 March 2023. The plants were acclimatized to the netted greenhouse conditions with bottom watering until the beginning of stress treatment. The details of the cultivation management procedure in the netted greenhouse were performed similarly to our previous study [28]. The air temperature and the temperature inside the netted greenhouse during the trial were shown in Supplementary Figure S5.

### 2.2. DNA Gel Blot Analysis

Genomic DNA was extracted from the transgenic and non-transgenic in vitro-grown poplar plants. Twenty µg of genomic DNA was fully digested by *Bam*HI or *Hin*dIII by overnight incubation. The resultant DNA fragments were electrophoresed, transferred to the nylon membrane, and hybridized with the DIG-labeled fragment of *HPTII* synthesized by the PCR reaction using a PCR DIG Probe Synthesis Kit (Roche, Basel, Switzerland). The signals were detected via chemical luminescence using Anti-Digoxigenin-AP, Fab fragments from sheep (Roche), and CDP-Star^®^ (Roche) by exposure for 140 min.

### 2.3. Reverse Transcription-Quantitative PCR (RT-qPCR)

For expression analysis, the total RNA was extracted from the leaves using the ISOSPIN Plant RNA (Nippon Gene, Toyama, Japan) kit following the manufacturer’s instructions. 0.5 µg of total RNA was used as a template for the reverse transcription using ReverTra Ace^®^ qPCR RT master Mix with gDNA Remover (Toyobo, Osaka, Japan). RT-qPCR was performed using THUNDERBIRD^®^ SYBR^®^ qPCR Mix (Toyobo) according to the following thermal cycle conditions: pre-incubation at 95 °C for 1 min, followed by 40 cycles of denaturing at 95 °C for 30 s, annealing and extension at 60 °C for 1 min, and a dissociation stage. The transcript level of *PtelF4a* (*Populus trichocarpa elongation factor 4a*) was used as reference for quantitation [27]. The primers used for RT-qPCR were as follows: 5′-CCACGAAAGAGTATGACGGC-3′ and 5′-AGCTTTGAGAATCCGACCGA-3′ for *AtSnRK2.8* and 5′-CAGTCTCTGCCACTCATGGA-3′ and 5′-GTGATCAGCACACGAGAGGA-3′ for *PtelF4a*.

### 2.4. Osmotic Stress Treatments

Osmotic stress treatments were conducted between 20 April and 20 May 2023. Aqueous solutions of polyethylene glycol 6000 (PEG, Fujifilm Wako Pure Chemical Corporation, Osaka, Japan) at 10% and 20% (*w*/*v*) concentrations were prepared and used to induce osmotic stress to the plants by bottom feeding. Plants treated with tap water instead of the PEG aqueous solution were used as the control. Each of the three lines and three treatments was provided with 3–4 individual plants.

### 2.5. Measurement of Photosynthesis Quantum Yields (QYs)

The photosynthetic quantum yields (QYs) in the leaves were monitored as a quantitative indicator of the damage caused by osmotic stress. Measurements of QYs were performed by FluoroPen-FP100 (Photon System Instruments, Drasov, Czech Republic). Qys were measured every evening when sunlight intensity or the amount of photosynthetically active radiation (PAR) was less than 50 µmol/m^2^/s. Qys were measured from the third, fourth, and fifth expanded leaves from the shoot apex of each of all individual plant. Three measurements were taken for each leaf, and the mean value was calculated.

### 2.6. Thermography Analysis of Leaf Surface Temperature

The measurement of the leaf surface temperature was performed using the testo 865-i thermal imager (Testo SE & Co., KGaA, Titisee-Neustadt, Germany). After watering, the potted plants were placed on a grid frame, and a thermal image was taken from above. The image was analyzed using testo IRSoft software (ver. 5.0.5628.36882, Testo).

### 2.7. Leaf Conductance

Stomatal conductance was measured using the Li-6400XT Portable Photosynthesis System (LI-COR, Inc., Lincoln, NE, USA) following the same measurement procedure described in the previous sections with a slight modification [29]. In brief, the light condition in the measuring head of the Li-6400XT instrument was set up to follow outdoor conditions, measured by the light-sensor connected to the instrument. The CO_2_ concentration was maintained at 400 ppm and the speed of the airflow was set to 500 μmol s^−1^. Observations four minutes after clamping the leaf to the instrument were used for analysis. Measurements were taken between 9 am and 1 pm. Leaves used for the evaluation varied from the third to the fifth leaf from the shoot apex depending on the availability of suitable-sized leaves on each of three plants for each of three lines and three treatments.

### 2.8. Analysis of Stomata from Leaf Imprints

Regarding the plants grown in the netted greenhouse, three intact leaves of about four-month-old plants were selected and used for taking leaf imprint. Since stomatal aperture differs during day and night cycles [30], collections were done both at night (11:30 p.m.) and during the daytime (8:30 a.m.). Regarding in vitro plants, intact leaves sampled from the plant subcultured in vitro in daytime were used for taking the leaf imprint. Observation of the stomata using leaf prints was performed using a widely known and common method [31]. Leaf imprinting was done by coating a thin layer of clear nail varnish on the abaxial surface of the designated leaves. The imprint was air-dried, peeled off from the leaf with transparent tape, and transferred to a glass slide.

The imprints were observed under a microscope (Olympus BX51) at 20× and/or 40× magnifications and images were captured with the assistance of cellSens software. Analysis of the acquired images was conducted using ImageJ software (ver. 1.53e, National Institute of Health, Bethesda, MD, USA).

The stomatal index (SI), defined as the ratio between the total number of stomata and the total number of epidermal cells, was determined and calculated using the formula:

SI = (number of stomata)/(number of epidermal cells)

The stomatal aperture (SA), the ratio between the minor and the major axis of each individual stoma, was also determined and calculated by the formula:

SA = (minor axis of stomata)/(major axis of stomata)

### 2.9. Statistical Analysis

Statistical tests by differences between groups were performed by non-parametric Mann-Whitney U test using the statannotations (v.0.6.0) on python environment (v.3.11.4). Other statistical tests by differences between groups were also performed by Tukey-HSD test based on Analysis of Variant analysis (ANOVA) using the multcomp package (v.1.4.25) on R environment (v.4.3.1). Graphs were generated using seaborn (v.0.12.2) on python environment.

## 3. Results

### 3.1. AtSnRK2.8 Transgenic Poplar Lines Showed Improved Tolerance to Moderate Osmotic Stress under Netted Greenhouse Conditions

Two genetically independent clonal transgenic lines, i.e., S01 and S22, overexpressing *AtSnRK2.8* were used for this study (Figure 1). It was confirmed that S01 has 1 copy of T-DNA containing the *AtSnRK2.8* expression cassette while the S22 has 2 copies (Figure 1b). The expression level of *AtSnRK2.8* in S22 was also higher than S01 (Figure 1). It could be considered that *AtSnRK2.8* driven by the CaMV35S promoter in the transgenic poplars were transcribing in whole body of plant independent from stress condition.

We previously attempted to evaluate the drought stress tolerance of the transgenic poplars under a 10-day irrigation suspension condition (Figure S6) but no significant difference was observed in the survival rates and quantitative damage indicators among the samples (Figure S6). Thus, we considered that the drought stress treatment performed in the previous experiment might be too severe to exhibit a significant difference in QY, as an indicator for plant healthiness between the non-transgenic plants (NT) but also for the two *AtSnRK2.8* transgenic lines (Figure S6). On the other hand, the data on the leaf surface temperature using a thermo-camera on the 5th day after the irrigation was suspended revealed that the leaf surface temperatures of *AtSnRK2.8* transgenic poplars were significantly higher than that of NT (Figure S7). We hypothesized that this observation was due to the differences in the transpiration rates caused by variations in stomatal apertures of the plants. Therefore, in this study, we adopted osmotic stress treatment using PEG solution watering to re-evaluate the stress tolerance of *AtSnRK2.8* transgenic poplars. The stress intensity for plants using this method was found to be easier to control compared to the previous irrigation suspension treatment.

**Figure 2:**
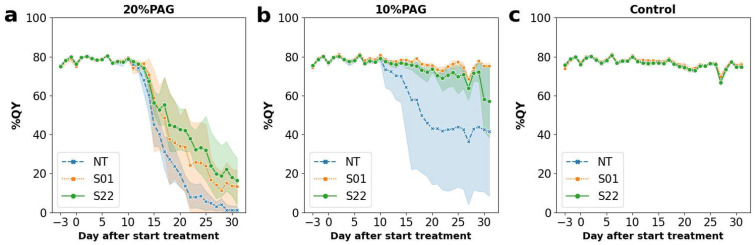
Fluctuations in QY during the stress treatments. Fluctuations in QY under 20% PEG, 10% PEG, and control treatments were shown in (**a**,**b**,**c**), respectively. Graphs were plotted by mean values of each measurement, and orange, green, and blue plots were shown that of transgenic S01 line and S22 line, and non-transgenic line (NT), respectively. Five individual plants were prepared for each combination of lines and treatments. Plots and shaded areas indicated mean and 95% confidence intervals, respectively.

Figure 2 shows the fluctuations of QY values during the period of stress treatment. In 10% PEG treatment, a clear decrease in QYs was observed only in NT, which eventually decreased to about 40% at 20 days after the start of treatment (DAT) (Figure 2b). On the other hand, in 20% PEG treatment, decreasing QYs were observed in all lines at 12 DAT (Figure 2a). The QYs of NT reached around 30% at 16 DAT and went below 10% on 21 DAT (Figure 2a). In contrast, QYs of the transgenic lines in 20% PEG treatment kept around 50% at 16 DAT and above 30% at 20 DAT. The QYs did not fall below 10% even after 30 days (Figure 2a). Comparing the two transgenic lines, QYs of the S22 line tended to be higher than that of the S01 line at 16 DAT (Figure 2a). In the control treatment, no changes in the QYs of any lines were observed throughout the period (Figure 2c). Similar results were obtained by observation of the SPAD value, which is an indicator for the chlorophyll content in leaves (Figure S8) [32]. These results – that both the QY and the SPAD values of *AtSnRK2.8* poplars maintained higher levels than that of the NT poplars during the osmotic stress treatments–suggested that *AtSnRK2.8* contributed to the alleviation of stress damages in poplar. On the other hand, the differences in the QY and SPAD values between the *AtSnRK2.8* transgenic and NT poplars were not statistically significant, suggesting that the direct contribution of *AtSnRK2.8* to improving stress tolerance was limited.

### 3.2. AtSnRK2.8 Affects Leaf Conductance of Poplar in Both Stress and Normal Conditions

**Figure 3:**
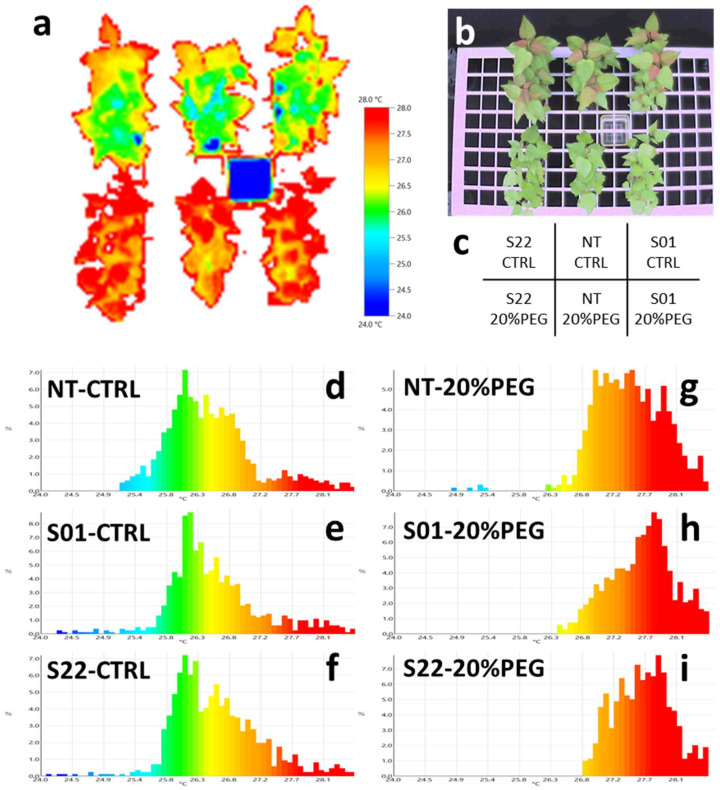
Leaf surface temperature on day 8 after start of the treatment. (**a**) The thermography image of transgenic and non-transgenic poplars in 20% PEG treatment and control, 8 days after the start of the treatments. (**b**) The bright image corresponding to the thermography image. (**c**) The sample layout of the thermography image. (**d**–**i**) Histograms showing temperature distribution for each combination of lines and treatments. The temperature distribution of the NT, S01, and S22 lines in the control treatment plots are shown in (**d**,**e**,**f**), respectively. The temperature distributions of the NT, S01, and S22 lines in the 20% PEG treatment experimental plots are shown in (**g**,**h**,**i**), respectively. Histogram colors match those in (**a**).

Stomatal closure prevents the cooling of the leaf by heat of evaporation, thus increasing leaf surface temperature. From our previous irrigation suspension experiment, we obtained preliminary results suggesting that *AtSnRK2.8* overexpression affects the regulation of stomatal aperture in poplars by leaf surface temperature observation (Figure S7). To further explore this observation, we also performed thermography analysis in this study. Figure 3 shows the temperature distributions of the leaf surfaces of the plants under the osmotic stress and control treatments at 8 DAT. There was no clear difference in the leaf surface temperature between transgenic and NT plants in non-stress, control conditions (Figure 3d–f). On the other hand, under the osmotic stress treatment, temperature distributions on leaf surfaces of transgenic poplars were higher than that of NT (Figure 3g–i). This result was consistent with the observation in the previous irrigation suspension treatments, confirming that leaf surface temperatures were higher in the *AtSnRK2.8* expressing transgenic poplars than in NT under osmotic stress conditions.

**Figure 4:**
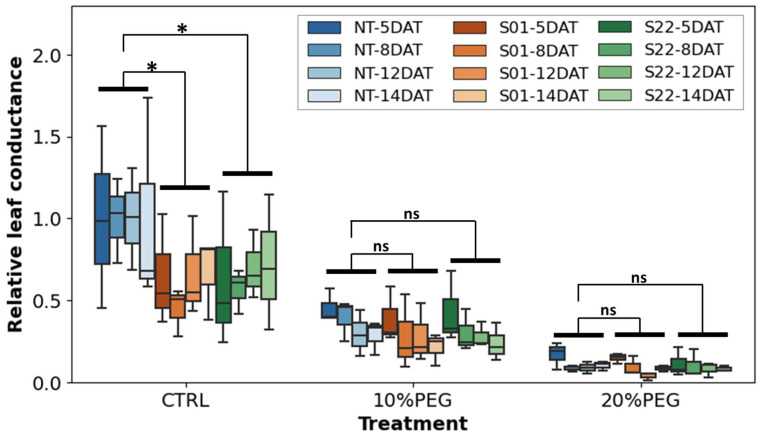
Leaf conductance during the stress treatments. Boxplots were shown leaf conductance measured at 5, 8, 12, and 14 days after the start of the treatments. Blue, orange, and green box plots show the leaf conductance of non-transgenic (NT), transgenic S01 line, and S22 line, respectively. Statistical tests were performed by the analysis of variance (ANOVA), and only combinations for which significant differences (α = 0.05) were detected were annotated with * in the graph (‘ns’ indicate no significance).

To further evaluate the expression of *AtSnRK2.8* and the effect of the stress treatment on stomatal aperture, leaf conductance of the plants under stress condition was measured (Figure 4). Under osmotic stress treatment, leaf conductance in all the treated plants was lower than the control, indicating that the osmotic stress induced stomatal closure. However, no clear difference was detected in the leaf conductance among transgenic and NT poplars under the osmotic stress treatments. Although, the median leaf conductance of transgenic plants in the 10% PEG plot tends to be lower than that of NT poplar. On the other hand, in the non-stress control group, the leaf conductance of the two transgenic plants, S01 and S22 lines, were significantly lower than that of NT (α = 0.01 and α = 0.05, respectively). These results suggested that the ectopic overexpression of *AtSnRK2.8* might have reduced the poplar leaf conductance independently of the osmotic stress treatment.

### 3.3. AtSnRK2.8 Might Affect Both Stomatal Index and Aperture in Poplar

Osmotic stress evaluation revealed that the transgenic poplars ectopically overexpressing *AtSnRK2.8* have altered stomatal behaviors in both stress and non-stress conditions. Therefore, we conducted a more detailed analysis on the state of the stomata of transgenic poplar under non-stress conditions by looking at their stomatal index and aperture through the leaf imprinting method.

**Figure 5:**
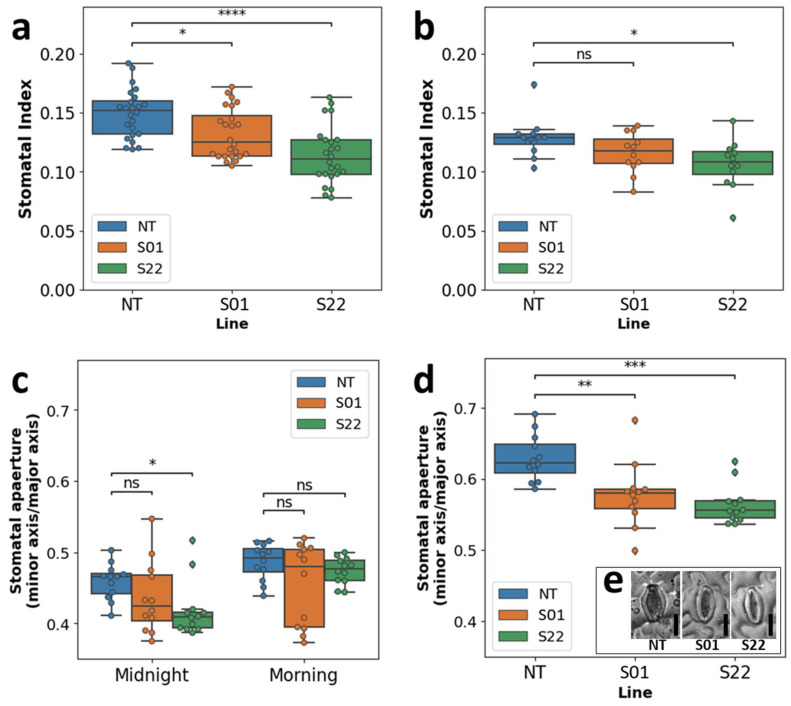
Stomatal index and aperture under the non-stress condition. Stomatal indexes of plants grown in the netted greenhouse and in vitro were shown as box plots and bee swarm plots in (**a**,**b**), respectively. The stomatal aperture of plants grown in the netted greenhouse and in vitro were shown as box plots and bee swarm plots in (**c**,**d**), respectively. Photos shown in (**e**) typical stomata of respective NT, S01, and S22 plant in vitro. Statistical tests were performed by Mann-Whitney U tests. *, **, ***, and **** indicate a significant difference (α = 0.05, 0.01, 0.001 and 0.0001, respectively) between respective samples, ‘ns’ indicate no significance (α = 0.05).

It was shown that the stomatal index of the two transgenic lines was significantly lower than that of NT (Figure 5a). In addition, no significant difference was observed in the stomata apertures among transgenic and NT plants during the morning (Figure 5c). However, the difference was observed at midnight wherein the stomatal aperture of S22 line was significantly lower compared to NT (Figure 5c). A verification of these differences was conducted using the in vitro-grown plants. It was confirmed that the stomatal index and aperture of the S22 transgenic poplar were significantly lower than that of NT even in the in vitro environment (Figure 5b,d). These results suggested that the ectopic overexpression of *AtSnRK2.8* reduces both the stomatal index and aperture in poplar.

## 4. Discussion

Drought stress has been shown to significantly reduce plant productivity and yield potential by up to 50% [33]. The United Nations Global Assessment Report on Disaster Risk Reduction (GAR) special report on drought in 2021 estimated a 6.4 billion USD direct loss because of drought in the US, 9 billion EUR in the European Union, and 18% fall in agricultural productivity in Australia between 2002–2010 due to the Australian Millennium Drought [34]. Furthermore, approximately three-quarters of the global arable area experienced a drought-induced yield loss over the period of 1983–2009 [35]. It’s not just crops but also trees that are affected by drought stress. Poplars are one of the most important forestry plantation trees and require a large amount of water resource, similar to or greater than field crops for their growth and survival [36]. Therefore, the improvement of drought-stress-tolerant plantation trees is an important research target. Novel knowledge regarding drought stress response in plants is crucial in developing climate-resilient trees. The research and development of a drought-stress-tolerant poplar using *Arabidopsis* genes involved in drought response and tolerance have been conducted [27,37].

The genus *Populus* generally exhibits poor water use efficiency and drought susceptibility. There is little knowledge on the molecular mechanisms of drought response in trees including poplars [38]. As such, our focus has been on SnRK2 kinases, which play an important role in the central pathway of ABA intracellular signal transmission in *Arabidopsis* under abiotic stress. Previous studies have demonstrated that *AtSnRK2.8* kinase is a key regulatory factor of stress response in *Arabidopsis*. Its overexpression enhances abiotic stress tolerance through transcriptional regulation [16]. In contrast, transgenic poplar overexpressing *AtSnRK2.8* only exhibited a slight increase in tolerance to abiotic stress [24,27]. Moreover, the transgenic poplar did not show significant transcriptional changes in abiotic stress responsive genes, and it was unclear how the transgenic poplar obtained the slight stress tolerance [24,27].

In this study, we evaluated the osmotic stress tolerance of poplars overexpressing *AtSnRK2.8* using PEG-6000 treatments in netted greenhouse conditions. These results show that both the QY and SPAD values of *AtSnRK2.8* poplars consistently kept in higher levels than that of the NT poplars during the stress treatments, suggesting that *AtSnRK2.8* contributed to the alleviation of osmotic stress damages in poplar (Figure 2 and Figure S7). It was also reported that the *AtSnRK2.8* poplars showed higher tolerances to osmotic and drought tolerance in laboratory conditions [27]. Both the results of the previous laboratory experiments and the greenhouse experiments in this study suggest that *AtSnRK2.8* poplars had higher stress tolerance traits than NT poplars. In *Arabidopsis*, it had been reported that *AtSnRK2.8*-overexpressing *Arabidopsis* plants exhibit extremely high stress tolerance because of constantly increased expression of ABA-responsive genes [16]. On the other hand, it has been reported that there is almost no change in the expression of ABA-responsive genes in the *AtSnRK2.8* poplar [27]. It means that the mechanism by which ectopic expression of *AtSnRK2.8* in poplar improved stress tolerance of poplar was unclear.

In this study, we also observed that the leaf conductance levels of the transgenic poplars were significantly lower than that of NT poplars (Figure 4). The observed reduction in stomatal conductance of the *AtSnRK2.8* poplars was also consistent with high leaf surface temperature of them (Figure 3, Figure 4 and Figure S7). These results are the first reports showing that *AtSnRK2.8* might be related to stomatal closure in poplars. To establish the cause behind this observation, we additionally explored the stomatal index and stomatal aperture in the leaves of the plants under non-stress conditions (Figure 5). The results showed that both the number of stomata and the stomatal aperture of the transgenic poplars were significantly lower than in NT poplars (Figure 5). This suggests that the overexpression of *AtSnRK2.8* may have affected both the stomatal aperture and differentiation in the poplar. These results could be considered to indicate that the improvement in stress tolerance of *AtSnRK2.8* poplars was due to a decrease in stomatal aperture and/or number.

The downstream outputs of subclass II and subclass III SnRK2 have been reported to have distinct different roles in *Arabidopsis*. Subclass III, and not subclass II, has been linked with control of stomatal aperture and differentiation [16]. Using loss-of-function mutants, Acharya et al. demonstrated that AtSnRK2.6 (OST1), an *Arabidopsis* subclass III SnRK2 kinase, is essential in ABA-dependent stomatal aperture control [39]. Recently, subclass III SnRK2 kinases were reported to control not only stomatal aperture but also stomatal differentiation by directly phosphorylating SPEECHLESS, the master transcription factor responsible for stomatal initiation [25]. However, the results of this present study suggested that AtSnRK2.8, a subclass II SnRK2 kinase, may have affected the stomatal aperture and differentiation in poplar.

**Figure 6:**
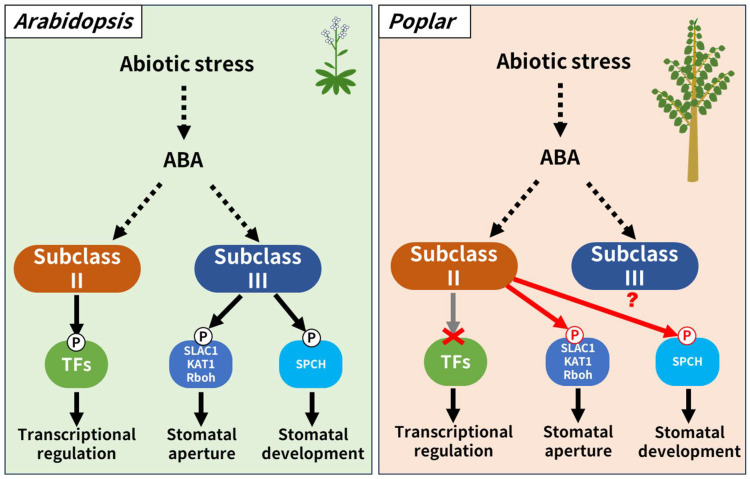
Scheme of a working hypothesis regarding the function of subclass II SnRK2 in poplar. In *Arabidopsis*, subclass II and subclass III SnRK2 regulate transcriptions and stomatal aperture/differentiation, respectively. It was previously reported that *AtSnRK2.8* is not involved in the expression of abiotic stress-responsive genes in poplar, but in this study, we revealed that *AtSnRK2.8* is involved in the differentiation and regulation of stomatal opening in poplar. Therefore, we proposed a working hypothesis that in poplar, unlike in *Arabidopsis*, subclass II SnRK2 outputs stomatal differentiation and regulation of opening. The function of subclass III SnRK2 in poplar is currently unclear.

As mentioned previously, subclass II SnRK2 targeted transcription factors that regulate stress-responsive gene expression in *Arabidopsis* rather than proteins associated with regulating stomatal behavior [40]. Moreover, it was known that subclass II SnRK2 outputs a stress-responsive gene expression as a downstream output, not only in *Arabidopsis*, a dicotyledonous herbaceous plant, but also in monocotyledonous herbaceous plants such as rice and wheat [41,42]. Thus, this present study first reported that subclass II SnRK2 kinase may regulate both stomatal aperture and differentiation in poplar. The reported phenotype of transgenic *Arabidopsis* plants overexpressing genes coding poplar subclass II SnRK2 kinase were very similar to that of the transgenic poplar overexpressing genes coding *Arabidopsis* subclass II SnRK2 kinases, which slightly restores abiotic stress tolerance without major changes in the transcriptome [22]. Based on these facts, we propose a working hypothesis that in sharp contrast to *Arabidopsis*, subclass II SnRK2 in poplar are involved in stomatal regulation as the downstream output (Figure 6). On the other hand, there is little experimental knowledge regarding the function of endogenous subclass II SnRK2 kinases in poplar which requires to be supplemented in the future. This is the same case for the endogenous subclass III SnRK2 kinases of the plant. It has been reported that the abiotic stress tolerance of *Arabidopsis* overexpressing poplar subclass III SnRK2 was not different from that of non-transgenic *Arabidopsis*, but there are no reports on the number of stomata or aperture [22]. We expect that the working hypothesis proposed here will be validated in the future as our understanding of endogenous subclass II and subclass III SnRK2 kinases in poplar increases.

## 5. Conclusions

Phenotypic evaluation of transgenic poplars ectopically overexpressing *AtSnRK2.8* suggests that subclass II SnRK2 kinases in poplar are involved in regulating both stomatal differentiation and aperture, as opposed to the transcriptional control reported in *Arabidopsis* studies. Stomatal regulation is known as an output of subclass III SnRK2 in *Arabidopsis*, but this new study suggests that in poplar, it is an output of subclass II SnRK2 kinases. Subclass II and subclass III SnRK2s exhibit different functions between *Arabidopsis* and poplar, in other words between herbaceous plants and woody plants. These findings are interesting from the perspective of the molecular evolution of the SnRK2 family, as well as a functional perspective and the possibility of utilization in biotechnology. There is little information on the signal transduction mechanisms underlying abiotic stress responses in trees. It is expected that future functional analyses on poplar SnRK2s will provide novel insights. Such findings will contribute to the development of abiotic stress-tolerant trees that are resilient and adaptable to climate change. Although a decrease in stomatal aperture prevents water loss, there is also concern that it may reduce the efficiency of photosynthesis and affect biomass productivity. Future evaluation through longer-term greenhouse and/or field cultivation trials will be required.

## Data Availability

The datasets used and/or analyzed during the current study are available from corresponding author on reasonable request.

## Supplementary Materials

The following supporting information can be downloaded at: https://www.mdpi.com/article/10.3390/life14010161/s1, Figure S1: Schematic model for classification of SNF1/AMPK/SnRK1 family; Figure S2: Phylogenetic tree of SnRK2s in *A. thaliana*, *P. trichocarpa* and *P. tremula*; Figure S3: Brief graphical representation of ABA controlled activity of SnRK2s, through PYR/PYL/RCAR–PP2C complex; Figure S4: Brief graphical representation of process of transgenic transformation of T89 poplars with *AtSnRK2.8* gene; Figure S5: Temperature condition inside of the netted greenhouse used for irrigation suspension and osmotic stress experiments; Figure S6: Fluctuations of QY during the irrigation suspension treatment; Figure S7: Leaf Surface temperature at 5 days after start of the irrigation suspension treatment; Figure S8: Fluctuations in SPAD values during the stress treatments.
