# Supplementary material for: Heterogeneous Expression of Arabidopsis Subclass II of SNF1-Related Kinase 2 Improves Drought Tolerance via Stomatal Regulation in Poplar
Source: Life (Basel). 2024 Jan 22;14(1):161. doi: 10.3390/life14010161 (PMC10817443; doi:10.3390/life14010161)
Supplement: Supplementary file 1 [file life-14-00161-s001.zip › life-2770286-supplementary.pdf]

Heterogeneous Expression of *Arabidopsis* Subclass II of SNF1-Related Kinase 2 Improves Drought Tolerance via Stomatal Regulation in Poplar

Borislav Horvat [1], Yuhei Shikakura [1], Misato Ohtani [2,3], Taku Demura [3,4], Akira Kikuchi [5,6], Kazuo N. Watanabe [5,6] and Taichi Oguchi [5,6,*]

[1] Degree Program in Life and Earth Science, Graduate School of Science and Technology, University of Tsukuba, Tsukuba 305-8572, Ibaraki, Japan
[2] Department of Integrated Biosciences, Graduate School of Frontier Sciences, The University of Tokyo, 5-1-5 Kashiwanoha, Kashiwa 277-8562, Chiba, Japan
[3] RIKEN Center for Sustainable Resource Science, 1-7-22 Suehiro-cho, Tsurumi-ku, Yokohama 230-0045, Kanagawa, Japan
[4] Center for Digital Green-Innovation, Nara Institute of Science and Technology, Ikoma 630-0192, Nara, Japan
[5] Institute of Life and Environmental Sciences, University of Tsukuba, Tsukuba 305-8572, Ibaraki, Japan
[6] Tsukuba Plant Innovation Research Center, University of Tsukuba, Tsukuba 305-8572, Ibaraki, Japan
* Correspondence: oguchi.taichi.ge@u.tsukuba.ac.jp

Supplemental Materials

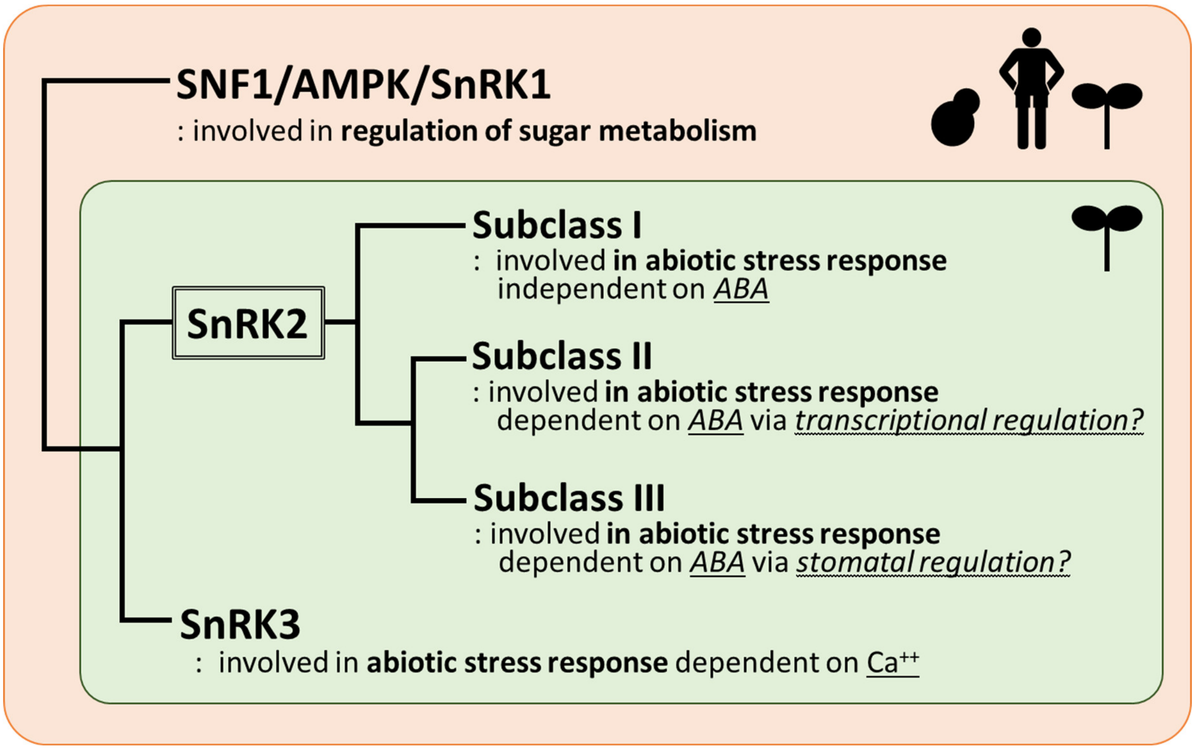

Figure S1. Schematic model for classification of SNF1/AMPK/SnRK1 family

SNF1/AMPK/SnRK1 family is a large family of kinases that is widely conserved from yeast to mammals and plants. However, SnRK2 and SnRK3 are subfamilies found only in plants.

SnRK2 is further categorized into ABA-independent subclass I and ABA-dependent subclass II and subclass III. The difference between subclass II and subclass III is the protein targeted for phosphorylation, with subclass II targeting transcription factors and subclass III targeting proteins involved in stomatal regulation, in *Arabidopsis*. SnRK3, like SnRK2, is involved in the regulation of stress responses, but in a calcium-dependent manner.

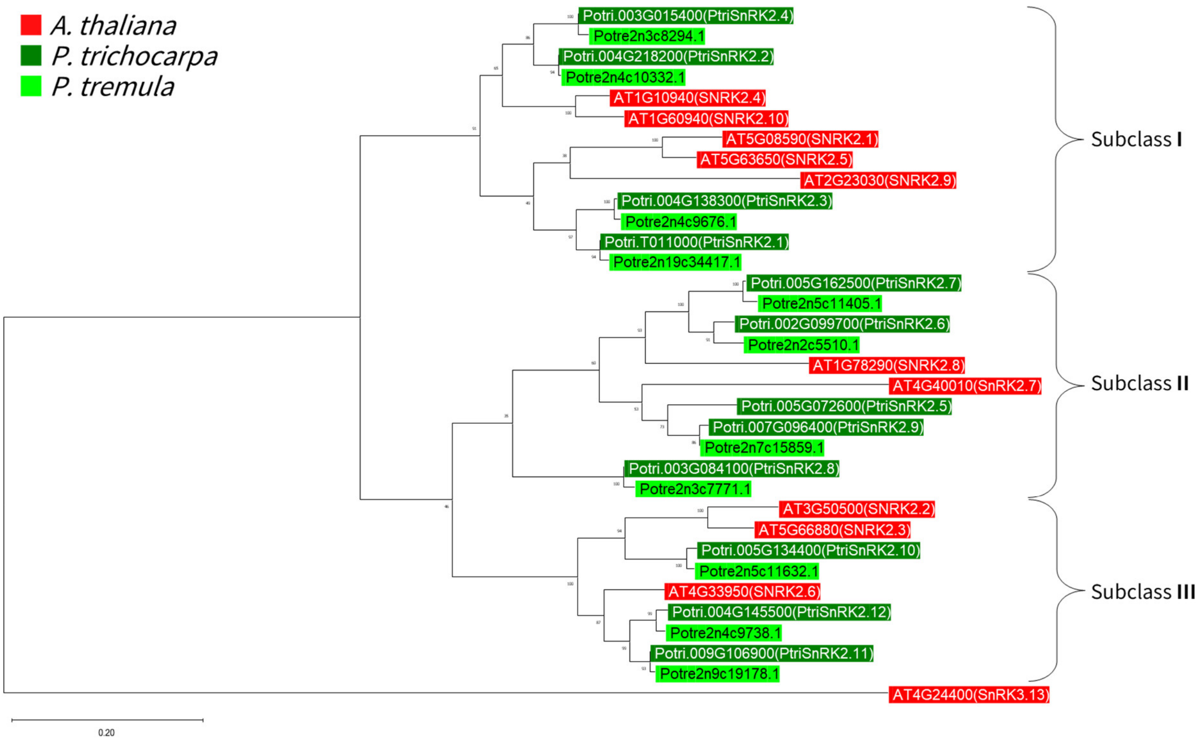

Figure S2. Phylogenetic tree of SnRK2s in *A. thaliana, P. trichocarpa* and *P. tremula*

Publicly available amino acid sequences of SnRK2 of *Arabidopsis thaliana* and two poplar species, i.e., *Populus trichocarpa*, and *P. tremula,* were used for analysis. AT4G24400, an *A. thaliana* SnRK3, was added as an outgroup. Amino acid sequences were clustered by interactive SALAD analysis on the SALAD Database site (https://salad.dna.affrc.go.jp/salad/10salad/salad.html). *A. thaliana*, *P. trichocarpa*, and *P. tremula* accessions are shown in red, green, and yellow green, respectively. The source of the amino acid sequence is shown below.

A. thaliana: The Arabidopsis Information Resource (https://www.arabidopsis.org/)

P. trichocarpa: Phytozome (https://phytozome-next.jgi.doe.gov/)

P. tremula: Plantgenie.org (https://plantgenie.org/)

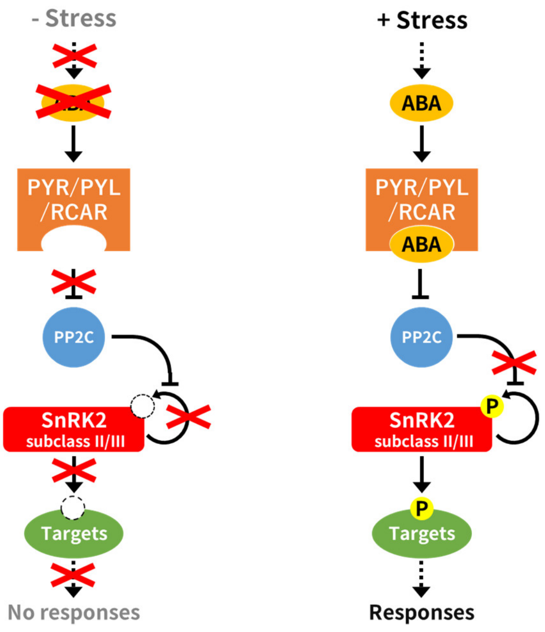

Figure S3. Brief graphical representation of ABA controlled activity of SnRK2s, through PYR/PYL/RCAR – PP2C complex.

Plants produce abscisic acid (ABA), a plant hormone, when they sense abiotic stress. Under normal conditions, phosphorylation activity of class II/III SnRK2 is suppressed by PP2C. Since activity of PP2C is inhibited by the complex of ABA and PYR/PYL/RCAR, phosphorylation activity of class II/III SnRK2 is not inhibited in the presence of ABA. Autophosphorylation of class II/III SnRK2, followed by phosphorylated activation of downstream target proteins, triggers stress responses. On the other hand, PYR/PYL/RCAR without ABA cannot inhibit activity of PP2C, and phosphorylation activity of subclass II/III SnRK2 is suppressed by PP2C, so no stress response occurs.

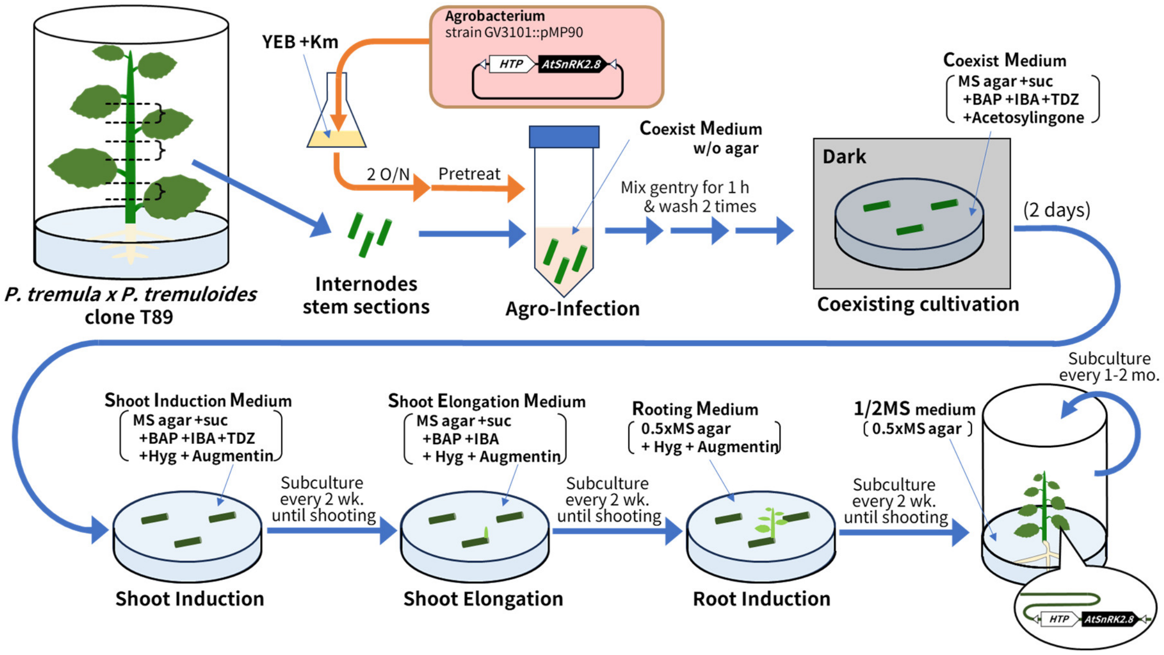

Figure S4. Brief graphical representation of process of transgenic transformation of T89 poplars with *AtSnRK2.8* gene

Internodal stem sections without apical and axillary buds are used as explants for transformation of hybrid aspen (*P. tremula* × *P. tremuloides*, clone T89). The explants are infected by being infiltrated for 1 hour with an Agrobacterium solution containing Ti-plasmid containing T-DNA as the transgene for plants. For the first 2 days after infection, explants are co-cultured in antibiotic-free medium in the dark. The explants are then placed on shoot induction medium. The explants that have formed shoots are then placed on a shoot elongation medium. Furthermore, explants with shoots that have grown to 1 cm or more are placed on a root induction medium. Rooted shoots are isolated from explants and become new candidates for transgenic plants. Plant cultures are subcultured every 2 weeks between shoot induction and rooting media, and every 1-2 months after transfer to 1/2 MS media.

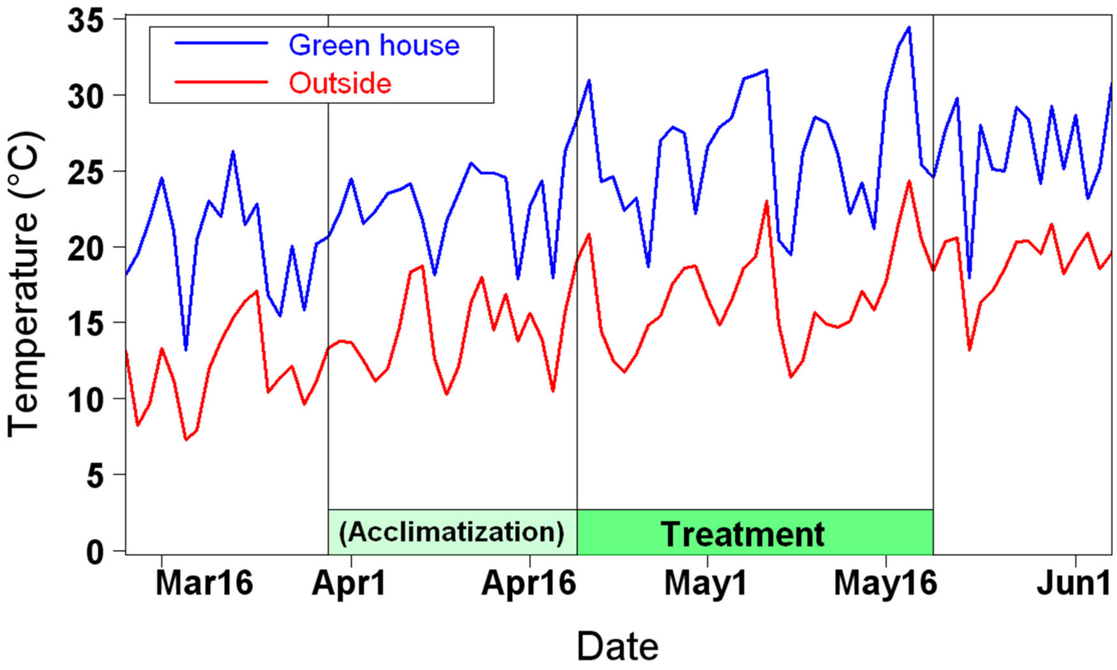

Figure S5. Temperature condition inside and outside of the netted greenhouse used for irrigation suspension and osmotic stress experiments.

Three-year average temperatures inside and outside of netted greenhouse for period from March 10 to June 1. Three-year average temperature in the netted greenhouse from 2020 to 2023 in the netted greenhouse is collected by thermo-logger, while three-year average outside temperature was monitored by AMeDAS post in Tsukuba/Tateno from 2020 to 2023.

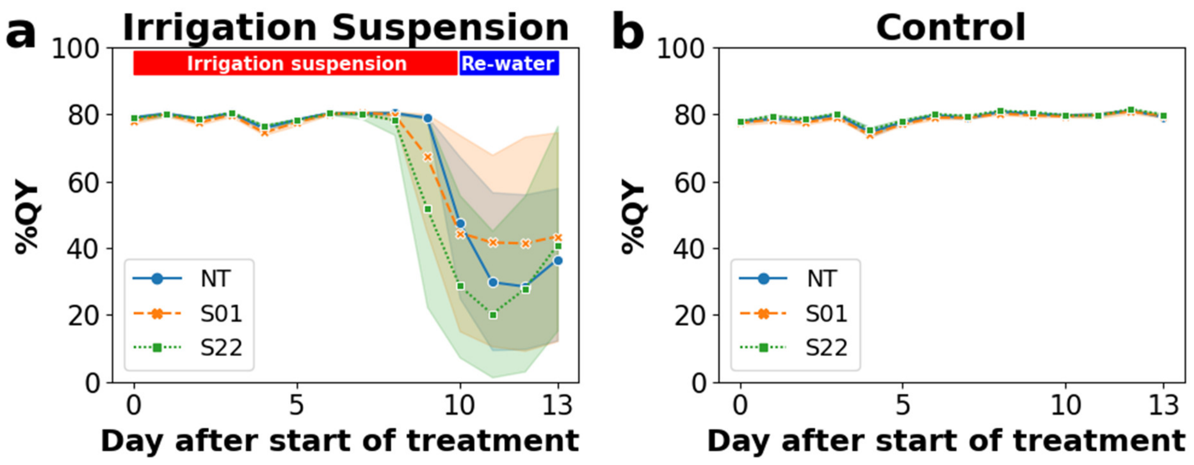

Figure S6. Fluctuations in QY during the irrigation suspension treatment

Fluctuations in QY of the two lines of *AtSnRK2.8* transgenic poplar (S01, S22) and non-transgenic poplar lines during a 10-day irrigation suspension treatment followed by a 4-day recovery treatment were shown in **a**. Graphs were plotted by mean values of each measurement, and orange, green, and blue plots were shown that of transgenic S01 line and S22 line, and non-transgenic line (NT), respectively. 5 individual plants were prepared for each combination of lines and treatments. Plots and shaded areas indicated mean and 95% confidence intervals, respectively. Fluctuations in QY of the plants maintained with optimal irrigation (control) during the same period were shown in **b**.

The plant materials and method for measuring QY was the same as that used in this study.

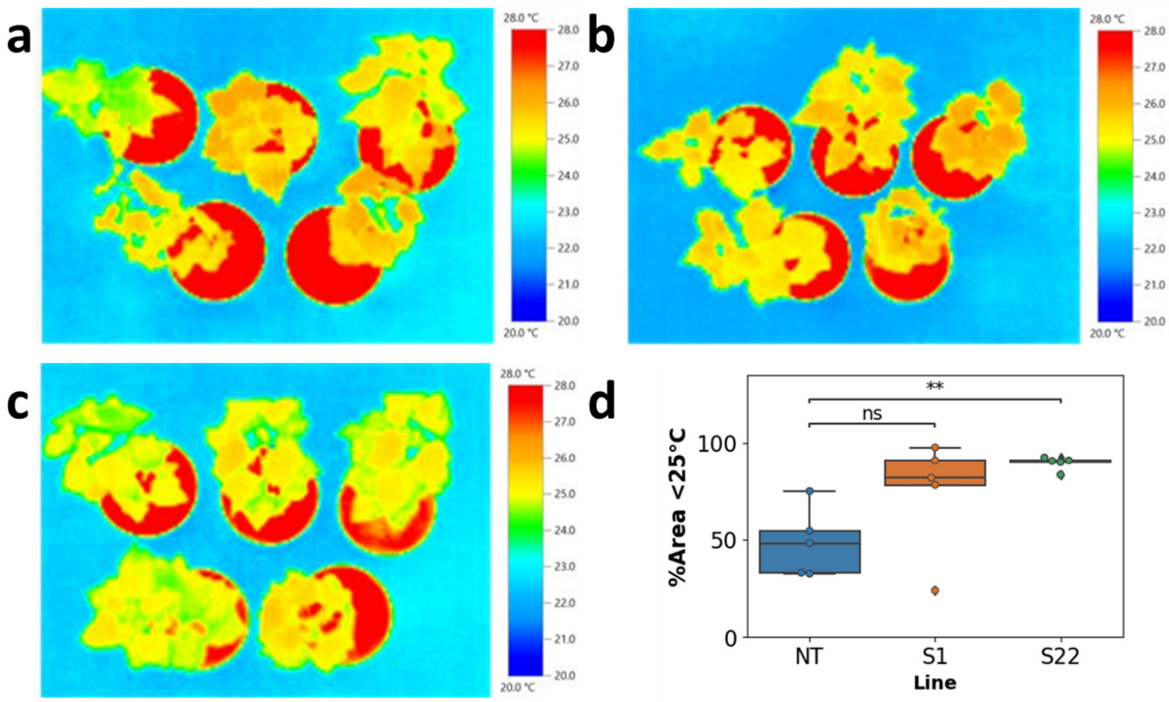

Figure S7. Leaf surface temperature at 5 days after start of the irrigation suspension treatment

Thermography images of the two lines of *AtSnRK2.8* transgenic poplar (S01, S22) and non-transgenic poplar lines taken at 5 days after start of the irrigation suspension treatment were shown in **a, b**, and **c**, respectively. Stomatal closure prevents cooling of the leaf by heat of evaporation, thus increasing leaf surface temperature. Box plot in **d** shows the percentage of leaf surface area where the surface temperature is 25°C or higher, calculated from the thermoimages in **a-c**. Statistical tests were performed by Tukey-HSD test. ** indicates significant difference (α= 0.01) between respective samples, 'ns' indicates no significance (α=0.05).

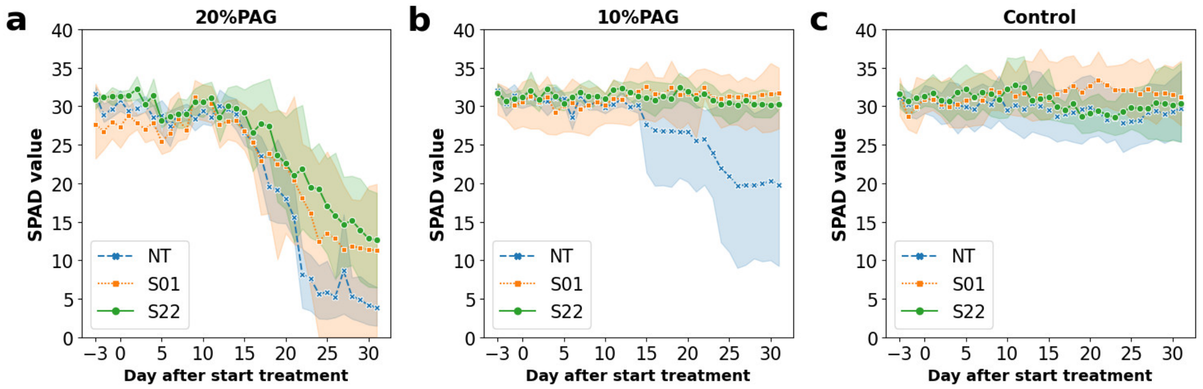

Figure S8. Fluctuations in SPAD values during the stress treatments Fluctuations in SPAD values under 20% PEG, 10% PEG, and control treatments were shown in A, B, and C, respectively. Graphs were plotted by mean values of each measurement, and orange, green, and blue plots were shown that of transgenic S01 line and S22 line, and non-transgenic line (NT), respectively. Shaded areas indicated 95% confidence intervals.

The SPAD values in leaves were monitored as quantitative indicator of damage caused by osmotic stress. Measurement of SPAD values was performed using SPAD-502 (Minolta Camera Co., Tokyo, Japan) on the same condition (i.e., time, leaf samples, number of measurements) as for the QY measurement method described in the main text of the article, chapter 2.4.1.
